# A Path to Precision Metabolic Treatment in Breast Cancer: Riluzole, Glutamate Signaling, and Invasive Lobular Carcinoma

**DOI:** 10.1210/jendso/bvad171

**Published:** 2023-12-26

**Authors:** Matthew J Sikora, Julie H Ostrander

**Affiliations:** Department of Pathology, University of Colorado Anschutz Medical Campus, Aurora, CO 80045, USA; Department of Medicine, Division of Hematology, Oncology, and Transplantation, University of Minnesota Medical School, Minneapolis, MN 55455, USA

**Keywords:** breast cancer, invasive lobular carcinoma, glutamate, metabolism, PET/CT, anti-estrogen

## Commentary

Invasive lobular carcinoma of the breast (ILC) presents distinct difficulties in diagnosis and clinical management. Detection is a particular challenge because ILC typically does not form a palpable mass and is often difficult to image, including by mammography and positron emission tomography/computed tomography (PET/CT). Recent studies indicate that PET/CT imaging with ^18^F-fluorodeoxyglucose (FDG), a mainstay in managing metastatic breast cancer, has limited utility in metastatic ILC [1]. A recent study by Olukoya and colleagues [2] offers insight toward leveraging this clinical liability into therapeutic opportunity.

FDG-PET/CT leverages the high avidity of most metastatic lesions for glucose and thus the FDG tracer. However, FDG uptake in metastatic ILC is limited, and many lesions are not detectable. In a head-to-head comparison of ^18^F-FDG with ^18^F-fluoroestradiol in 7 patients with estrogen receptor α (ER)-positive ILC, 268 bone lesions were detected by either method, with 94% being ^18^F-fluoroestradiol-positive but only 34% being FDG positive [1]. This limited FDG avidity suggests that ILC rely less on glucose than other breast cancers; several studies to date support that ILC has a distinct metabolic phenotype [3-6], yet studies of how ILC may differentially utilize or rely on other fuels like fatty acids or amino acids are in their early stages. Understanding and ultimately leveraging the unique metabolism of ILC is important in better understanding ILC etiology and improving patient care.

Olukoya et al build off prior work from the Riggins Laboratory identifying that metabotropic glutamate receptors (GRM), ie, G-protein coupled receptors activated by extracellular glutamate, contribute to antiestrogen resistance in ILC cells [7]. Stires et al showed that targeting GRM with riluzole improved response to the antiestrogen fulvestrant in the endocrine-resistant ILC model LCCTam (derived from SUM44PE). Riluzole is a Food and Drug Administration-approved treatment for amyotrophic lateral sclerosis that inhibits glutamate release and is used to starve GRM of extracellular glutamate and/or directly inhibit GRM. Targeting glutamate signaling is intriguing in ILC in part due to clinical imaging data that contrasts the shortcomings of FDG-PET; ^18^F-fluciclovine, an animo acid analog imported into cells via glutamine transport and thus glutamate metabolism, is readily taken up by ILC [8]. In the current study, Olukoya et al expand the observations of riluzole efficacy in LCCTam to multiple ILC and antiestrogen-resistant ILC models (in vitro and in vivo), compare efficacy to models from invasive ductal carcinoma (ie, breast cancer of no special type), and test riluzole in patient tumor explant cultures. Notably, since ∼95% of ILC are ER-positive, comparing riluzole to fulvestrant or the combination is a key feature of the study. These new data support that riluzole indeed has a distinct impact on ILC cell proliferation and survival compared to IDC that should be further developed to target and exploit metabolic signaling in ILC. Importantly, these data also highlight that the cross-talk between ER signaling and metabolic signaling may confound efforts to target ILC metabolism, despite antiestrogens being central to ILC treatment, and that antiestrogen resistance may create distinct metabolic states that will be differentially responsive to metabolic inhibitors.

Riluzole treatment caused an ILC-specific cell cycle arrest in G2/M, which was observed in 3 ER+ ILC cell lines and 2 associated antiestrogen-resistant variants, contrasting a G0/G1 arrest in MCF7 (no special type) and nontransformed MCF10A cells. Cell cycle arrest was associated with an increase in apoptotic cells in 1 of the parental/anti-estrogen resistant ILC pairs. Importantly, the observation that riluzole induced cell cycle arrest in both antiestrogen-resistant ILC models tested suggests that targeting GRM may be effective in both ER-dependent and -independent settings, as LCCTam and MM134:LTED were previously shown to be fulvestrant responsive and resistant, respectively. Riluzole also had single-agent efficacy against HCI-013EI (estrogen-independent ILC) patient-derived xenograft in vivo growth, with 3/5 tumors growth-suppressed and exhibiting an increase in caspase-3-mediated apoptosis. Despite this potential mechanistic separation from ER, riluzole and fulvestrant combined showed minimal additive effect on growth suppression in vitro, and the combination also had limited increase in efficacy over fulvestrant alone against HCI-013EI in vivo growth. Future studies will need to consider the disparate cell cycle effects of fulvestrant and other antiestrogens, which primarily cause G0/G1 arrest, vs the ILC-specific G2/M arrest caused by riluzole and whether the former antagonizes apoptotic effects of the latter. Similarly, in causing cell cycle arrest, antiestrogens suppress and remodel metabolism in ER+ breast cancer cells including ILC cells [6], which may limit metabolic demands and potentially the efficacy of blocking metabolic signaling. Careful sequencing may be particularly critical for combining riluzole or related drugs with antiestrogens. Though regulation of glutamate signaling by ER or antiestrogens in ILC remains to be directly studied and is an important future direction, fluciclovine uptake was reduced by neoadjuvant chemotherapy in ILC [8], supporting that at least chemotherapy can suppress glutamate signaling. However, riluzole combined with fulvestrant clearly merits further investigation in ILC—in the patient tumor explant studies, combination treatment increased apoptosis over single agent specifically in the ILC tumor among the 5 explants tested and not the other breast tumors.

Substantial clinical and laboratory data, in particular clinical imaging studies, support that ILC has a distinct metabolic phenotype wherein tumors are likely less reliant on glucose and more reliant on other metabolic pathways. This new study from Olukoya et al and the Riggins Laboratory builds key insight that targeting glutamate signaling with riluzole has distinct impacts on ILC cells relative to other breast cancer cells, which can form a foundation for precision treatments targeting ILC metabolism. Future studies will need to address the undoubtedly complex interplay between cell metabolism, ER signaling, and antiestrogen response and resistance. A better mechanistic understanding of GRM signaling and glutamate metabolism in ILC has the potential to lead to ILC-tailored combination treatments targeting ILC-specific metabolism and can identify other related metabolic vulnerabilities in ILC.
